# Genetic diversity of hepatitis E virus (HEV) in imported and domestic camels in Saudi Arabia

**DOI:** 10.1038/s41598-022-11208-6

**Published:** 2022-04-29

**Authors:** Sherif A. El-Kafrawy, Ahmed M. Hassan, Mai M. El-Daly, Mohammed Al-Hajri, Elmoubashar Farag, Fatimah Ahmed Elnour, Anas Khan, Ahmed M. Tolah, Thamir A. Alandijany, Noura A. Othman, Ziad A. Memish, Victor M. Corman, Christian Drosten, Alimuddin Zumla, Esam I. Azhar

**Affiliations:** 1 Special Infectious Agents Unit-BSL3, King Fahd Medical Research Center, King Abdulaziz University, Jeddah, Saudi Arabia; 2 Department of Medical Laboratory Sciences, Faculty of Applied Medical Sciences, King Abdulaziz University, Jeddah, Saudi Arabia; 3 Department of Medical Laboratory Technology, Faculty of Applied Medical Sciences, King Abdulaziz University, Rabigh, Saudi Arabia; 4 Ministry of Public Health, Doha, Qatar; 5 The Global Centre for Mass Gatherings Medicine, Public Health Directorate, Ministry of Health, Riyadh, Saudi Arabia; 6 King Saud Medical City, Ministry of Health, Riyadh, Saudi Arabia; 7 Al-Faisal University, Riyadh, Saudi Arabia; 8 Hubert Department of Global Health, Rollins School of Public Health, Emory University, Atlanta, GA USA; 9 Charité-Universitätsmedizin Berlin, Freie Universität Berlin, Humboldt-Universität Zu Berlin, Berlin, Germany; 10 Berlin Institute of Health, Institute of Virology, Berlin, Germany; 11 Department of Infection, Division of Infection and Immunity, Centre for Clinical Microbiology, University College London, London, UK; 12 NIHR Biomedical Research Centre, University College London Hospitals, London, UK

**Keywords:** Infection, Microbiology, Pathogenesis

## Abstract

Camels gained attention since the discovery of MERS-CoV as intermediary hosts for potentially epidemic zoonotic viruses. DcHEV is a novel zoonotic pathogen associated with camel contact. This study aimed to genetically characterize DcHEV in domestic and imported camels in Saudi Arabia. DcHEV was detected by RT-PCR in serum samples, PCR-positive samples were subjected to sequencing and phylogenetic analyses. DcHEV was detected in 1.77% of samples with higher positivity in domestic DCs. All positive imported dromedaries were from Sudan with age declining prevalence. Domestic DcHEV sequences clustered with sequences from Kenya, Somalia, and UAE while imported sequences clustered with one DcHEV isolate from UAE and both sequences clustered away from isolates reported from Pakistan. Full-genome sequences showed 24 amino acid difference with reference sequences. Our results confirm the detection of DcHEV in domestic and imported DCs. Further investigations are needed in human and camel populations to identify DcHEV potential zoonosis threat.

## Introduction

Approximately 60% of all human pathogens and 75% of emerging infectious diseases are zoonotic (of animal origin). Contact with Dromedary camels (DCs) or their products was identified as major risk factor for zoonotic spillover to humans of the novel lethal coronavirus, Middle East Respiratory Syndrome Coronavirus (MERS-CoV)^1–4^. MERS-CoV remains in the WHO Blueprint list of priority pathogens. Since the first discovery of MERS-CoV, increasing attention is being focused on dromedary camels (DCs) as intermediary hosts for novel zoonotic viruses with epidemic potential.

Several recent reports of possible transmission of Hepatitis E Virus (HEV) to humans through consumption of animal products and/or frequent contact with animals have led to the acknowledgement of HEV as an important emerging zoonotic pathogen of humans of global public health significance^5^. HEV is a member of the family *Hepeviridae,* genus *Orthohepevirus A*. The virus is a common cause of acute viral hepatitis in humans, causing up to 20 million human infections annually. HEV is one of the leading causes of illness in the developing world, is endemic in Central Asia, with outbreaks reported from the Middle East and Central America. HEV has relatively high mortality rates among pregnant women and young children^6,7^. Chronic HEV infection can cause more severe disease in immunocompromised individuals, such as people living with HIV, organ transplants and other immunosuppressivestates^8,9^.

There are four main genotypes, HEV1, HEV2, HEV3 and HEV4^10^. HEV1 and HEV2 infect only humans. HEV is shed in the stools of infected persons and is transmitted in areas with poor sanitation through contaminated water supplies. In areas with good sanitation and pure water supplies, animals are a major source of HEV infection to humans, human to human transmission is also reported through blood transfusion^11^. HEV has been identified in a wide range of mammals including pigs, boar, deer, rodents, ferrets, rabbits, mongoose, bats, cattle, sheep, foxes, minks, and horses^12–15^. HEV3 and HEV4 circulate in several animals such as pigs, wild boar, deer, other mammals and infect humans through consumption of contaminated raw or undercooked pork or game meat^16^. Several studies have reported serological evidence of HEV in blood donors, hemodialysis patients and blood transfusion recipients^17–20^. A novel HEV genotype, named Dromedary camel hepatitis E virus (DcHEV), was initially detected in dromedary camels (DCs) in the Middle East in 2014^21^. DcHEV was first reported to cause chronic hepatitis in a transplant patient in UAE who regularly consumed camel milk and meat^22^. Recent studies from UAE have confirmed the presence of DcHEV-specific viral genomes in DCs^23^.

In Saudi Arabia and other Middle Eastern communities, camels serve considerable social and economic roles since they are used as domestic pets, reared for their milk and meat, racing and beauty contests. Saudi Arabia is the world’s largest importer of DCs from African countries, while countries of the horn of Africa are the main exporter of camels to the Arabian Peninsula^2,24^. In Saudi Arabia, we recently showed that the prevalence of DcHEV antibodies in domestic camels is slightly higher than in camels imported from Africa^25^. Identifying DcHEV genotypes and performing detailed molecular and phylogenetic characterization of the virus in DCs is critical for surveillance, understanding the evolution of human infections and designing appropriate control and preventive measures. Therefore, we performed this molecular and phylogenetic analyses of DcHEV in domestic and imported camels in Saudi Arabia.

## Materials and methods

### Ethical statement

Ethical permission to conduct this study was obtained from the Directorate of Agriculture, Ministry of Environment, Water and Agriculture, Jeddah, Saudi Arabia. The study was approved by the Unit of Biomedical Ethics, King Abdulaziz University Hospital, (Approval number 16-121). All methods and procedures in this study were carried out in accordance with ARRIVE guidelines and regulations.

### Dromedary camels

Two cohorts of DCs were enrolled in this study. The first cohort comprised DCs imported into Saudi Arabia on incoming ships from Sudan or Djibouti at the Sea Port of Jeddah. Samples were collected over a period of 2 years from 2017 to 2019. The second cohort comprised local DCs in Jeddah, Saudi Arabia and were collected from three camel farms and from an abattoir in the city before camels were slaughtered.

All methods were carried out in accordance with relevant guidelines and regulations.

### Sample collection

Jugular blood samples were collected from both cohorts of DCs. The blood samples were left to clot and then centrifuged, serum obtained and stored at − 80 °C till testing.

### RNA extraction and HEV RT-PCR

Serum samples were pooled in 9 samples per pool. RNA was extracted from serum pools using the Qiaquick viral RNA extraction kit (Qiagen, Germany) according to manufacturer recommendation. Extracted RNA was subjected to real time RT-PCR using the LightMix^®^ Modular Hepatitis E Virus (HEV) (TIB MOLBIOL, Germany). Samples in positive serum pools were tested separately to identify the positive individual sample using the same protocol.

### Sequencing and phylogenetic analysis

Positive individual samples were subjected to nested RT-PCR reaction as described earlier^26,27^. In brief, a ~ 341 bp fragment of the HEV RNA dependent RNA polymerase (RdRp) gene was amplified and the resulting PCR product was purified using the agarose gel purification kit (Qiagen, Germany) and subjected to direct sequencing using the BigDye dideoxy termination kit on the ABI 3500 sequence analyzer (Applied Biosystems, Germany). The full genome sequencing was performed on two positive cases one imported and one domestic case. The full genome sequence was performed according to Lee et al.^22^. The full HEV genome was amplified in 19 fragments and the PCR products were purified from agarose gel and sequenced using the same protocol as for the RdRp fragments. The fragments were assembled using the assembly tool of the Geneious prime software (version 2021.12.2)^28^. The generated sequences were multiple aligned using Geneious software and phylogenetic trees were produced using the maximum likelihood method with 1000 bootstrap replicates. Sequences were deposited in Genbank and were assigned the accession numbers MZ027568–MZ027584 for the RdRp gene sequences and MW835252–MW835253 for the two full genome sequences.

## Results

### Camels and blood sample collection

Blood samples were collected from 1189 DCs: 893 (75.1%) were from DCs imported from Africa (724, from Sudan and 169 from Djibouti). Two hundred and ninety-six were domestic Saudi DCs from local farms and slaughterhouses in and around Jeddah. 1085 out of 1189 DCs were male (91.3%) and 104/1,189 (8.7%) were female. Nine hundred and fifty two out of 1189 (80.1%) DCs were 3–5 years old. 229 (19.2%) were between 1 and 3 years of age and 8 (0.7%) were < 1-year-old.

### RT-PCR DcHEV RNA testing

RT-PCR testing of the samples showed DcHEV RNA in 21/1189 camels (1.77%) with a higher prevalence of DcHEV RNA in domestic dromedaries (12/296, 4.1%) as compared to the imported dromedaries (9/893, 1.0%), Ct values from the real time RT-PCR assay are shown in Supplementary Table 1. All positive cases were male dromedaries and all the positive imported dromedaries were imported from Sudan. The highest prevalence was found in the juvenile dromedaries < 1 year (1/8, 12.5%), followed by the age group 1–3 years (9/229, 3.9%), while the lowest prevalence was found in the age group 3–5 years (11/952, 1.2%).

### Sequencing and phylogenetic analyses

Positive PCR samples were subjected to direct sequencing, and we were able to sequence 19 out of 21 (90.5%) positive samples in the RdRp gene. Phylogenetic analysis of the generated sequences showed that all sequences belong to HEV genotype 7. They clustered with sequences reported earlier mainly from UAE and Kenya. The RdRp sequences showed mutations at 95 nucleotide positions with 6 non-synonymous mutations leading to amino acid changes (Supplementary Table 2) with more frequency in domestic compared to imported dromedaries. The RdRp showed higher similarities among imported dromedary sequences compared to domestic sequences (Supplementary Fig. 1).

The full genome sequences showed clustering with the two available full genome sequences in the Genbank. They showed 242 nucleotide mutations including 24 nonsynonymous mutations that led to amino acid changes (Supplementary Table 3) along the viral genome when compared to the reference sequence KJ496143-HEV-7a.

**Figure 1:**
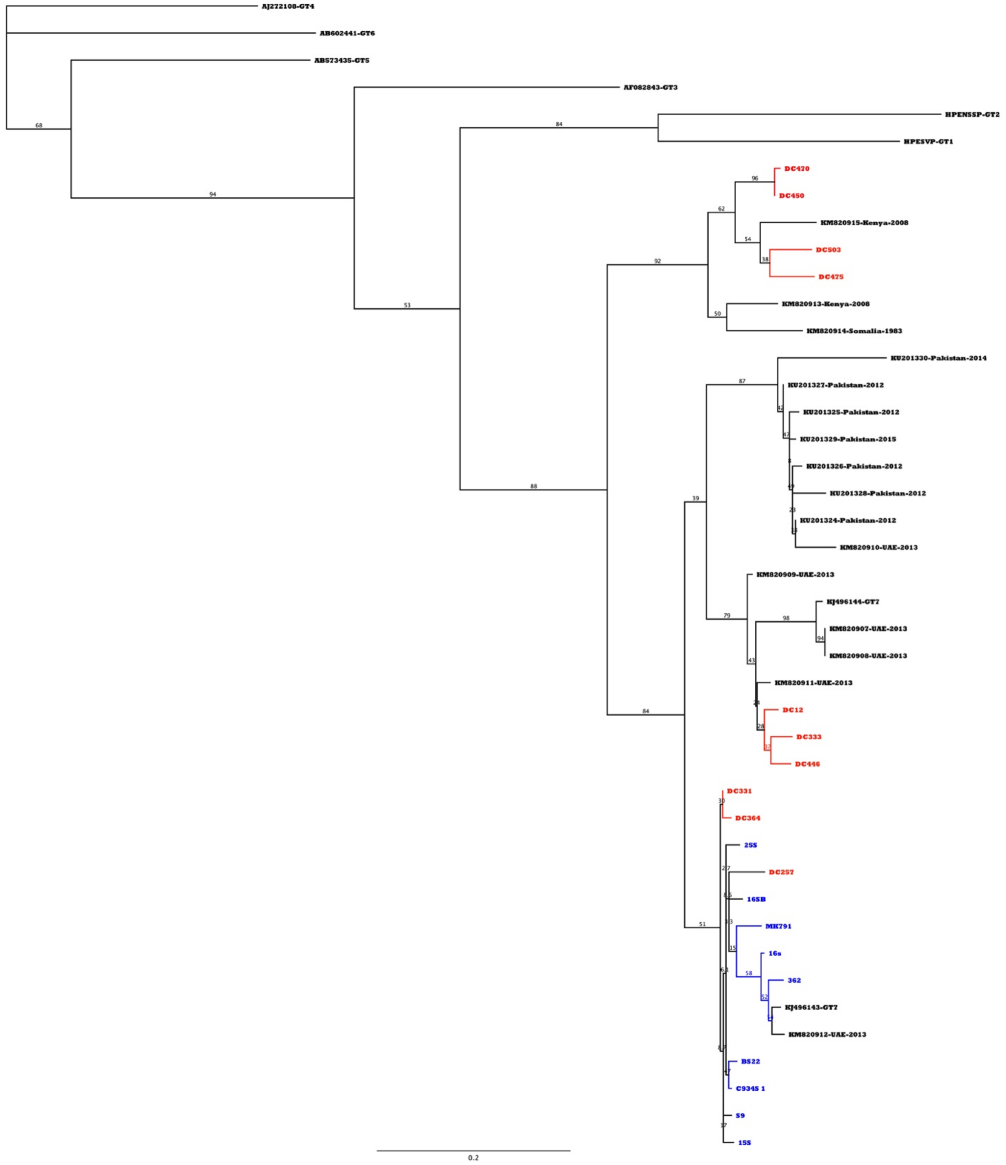
Phylogenetic tree for the RdRp gene of DcHEV Red-colored sequences represent domestic camels while blue colored sequences represent imported sequences. Phylogenetic tree was constructed using Maximum Likelihood method with 1000 bootstrap replicates using Geneious prime software (version 2021.2.2).

Figure 1 shows the grouping of all sequencing with the reference sequences of HEV genotype 7 (KJ496143 and KJ496144) together with the previously reported GT-7 sequences by Rasche et al.^26^. Phylogenetic tree also shows the clustering of the sequences mainly with the African sequences and sequences from UAE but not with sequences from Pakistan.

**Figure 2:**
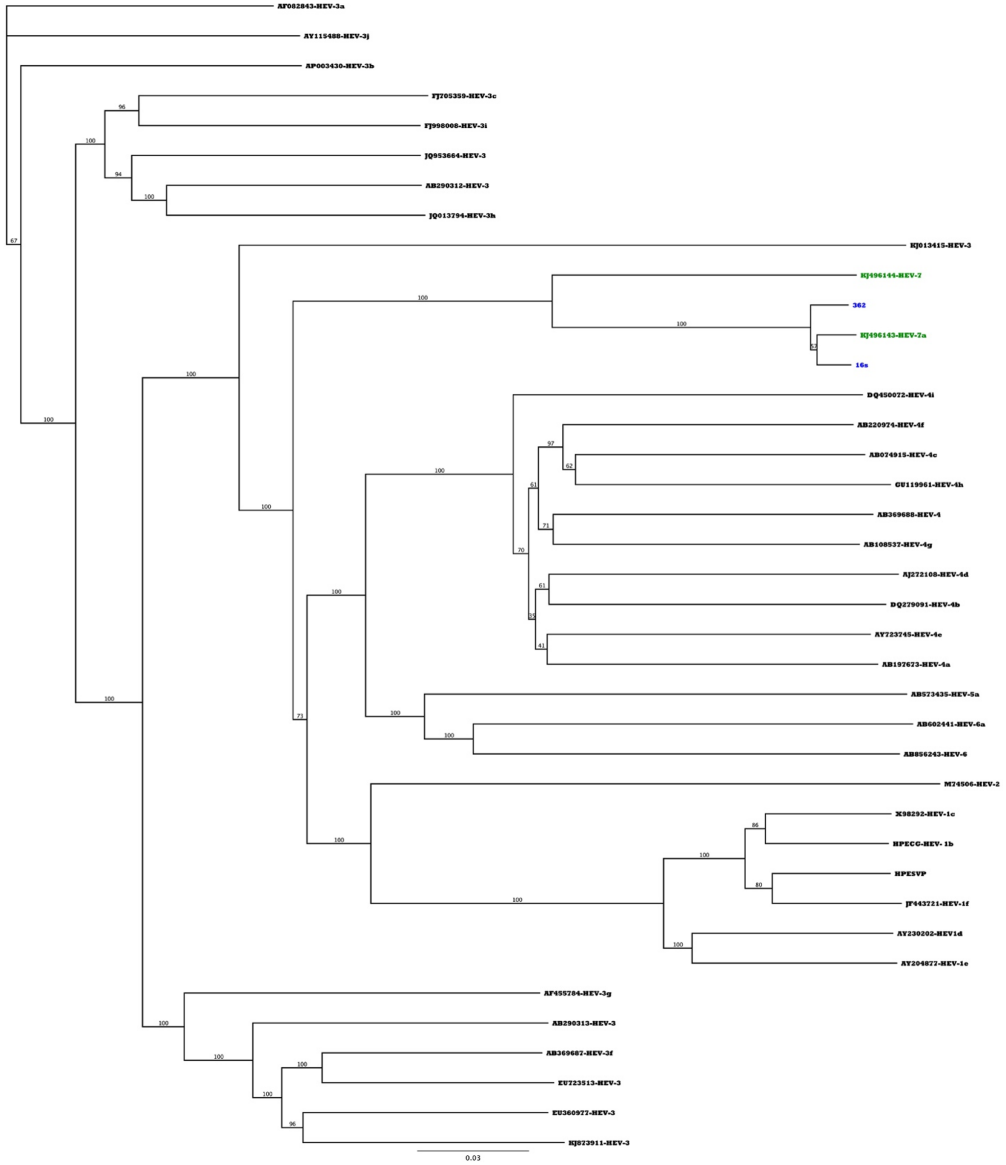
Phylogenetic tree for the two full genome sequences generated in the study. Blue-colored labels represent the new sequences from this study, while the blue-colored labels represent the two full genome reference sequences available in the Genbank. Phylogenetic tree was constructed using Maximum Likelihood method with 1000 bootstrap replicates using Geneious prime software (version 2021.2.2).

Figure 2 shows the clustering of the two full genomes generated from this study with the two available genotype 7 from Genbank.

## Discussion

Our data add further to the growing knowledge on the role of HEV as a possible camel associated zoonotic pathogen with epidemic potential. There are several notable findings from our study.

*First*, our study of DcHEV in domestic and imported DCs from Africa in Saudi Arabia is the largest published to date and provides evidence for the presence of DcHEV in both domestic and imported camels from Africa with evolving genomic changes.

*Second*, phylogenetic analysis of the RdRp sequences identified infection with HEV genotype 7. When comparing with HEV genotype 7 sequence KJ496144, the two full genome sequences from our study showed 242 nucleotide variations including 24 nonsynonymous mutations leading to the amino acid changes.

*Third*, sequences from domestic camels formed a separate cluster together with published sequences from Kenya, Somalia, and UAE.

*Fourth,* RdRp sequences of imported camels clustered with one DcHEV isolate previously reported from UAE. Both domestic and imported sequences clustered away from isolates reported from Pakistan. The higher number of mutations in the RdRp sequences of domestic dromedaries compared to imported dromedaries indicate a potential multiple sources of infection but this needs more studies on larger sample numbers to be confirmed. To have solid evidence of the source of HEV infection in dromedary camels in Saudi Arabia a larger number of full genome sequences need to be generated from both domestic and imported dromedaries and phylogenetically analyzed. The low volume of our serum samples did not allow for the generation of more than two full genome sequences.

*Fifth,* full-genome sequences generated from this study were added to the only two full-genome sequences of genotype 7 in GenBank. The analysis showed a 24 amino acid difference between our sequences and the GenBank sequences while the other 218 nucleotide mutations were synonymous mutations and did not result in amino acid changes.

*Sixth*, we found that domestic DCs from Saudi Arabia had higher prevalence of DcHEV RNA than imported camels from Africa.

HEV is a cause of morbidity in several regions around the world, where it is endemic in some developing countries with poor sanitation/hygiene and frequent water contamination, which are the most common risk factors^29^. While in developed countries like the US, Japan and Europe, the most common risk factor is the consumption of infected meat and animal products^30^. The zoonosis of HEV was established by the identification and characterization of HEV isolated from pigs that have close genetic relationship to human HEV^31^. Several other reports on the transmission of HEV to human through consumption of animal products or frequent contact with animals have led to the acknowledgement of HEV as an important human zoonosis^5^. HEV studies from Saudi Arabia have been scanty. One seroprevalence study in 2002 reported HEV seroprevalence ranging from (7.2% vs. 10.8%) in dialysis patients from Jeddah as compared to 16.9% and 18% HEV seroprevalence in blood donors in Jeddah (West)^19^ and sickle cell anemia in children from Jizan (South)^17,18^, respectively.

The importance of investigating HEV infection in camels in Saudi Arabia comes from the social and economic role that dromedaries play in the Arabian Peninsula. Dromedaries constitute a source of income through camel trade, camel races and camel shows and consumption of meat and milk. Camel herds frequently move across the Arabian Peninsula and might cross the borders of many Gulf countries for many reasons such as grazing, participation in camel races and camel shows. Camels’ frequent movement might increase the risk of transmitting the infection to different populations of these countries. However, there is no official national identification procedures nor obligatory vaccination campaigns focused on camels; it is difficult to quantify the extent of these movements. Camels from both UAE and Qatar travel every year during the winter seasons (November–February) for grazing, shows and racing in the Eastern region of Saudi Arabia.

The main source of dromedary camels to the Arabian Peninsula is the importation of life camels from the Horn of Africa (Sudan, Djibouti, Somalia, Ethiopia and Kenya) to the Arabian Peninsula. The importance of performing this study in Jeddah comes from the geographical location of the seaport in Jeddah as the main port of entry of dromedaries from Africa^32^. In 2013, official records in Saudi Arabia reported the import of 131,932 camels representing more than 70% of the animals slaughtered in the country^33^. Detailed spatiotemporal studies of camel movement patterns are lacking and are urgently needed to try and understand the role of camel movement in spreading the infection.

Recent studies from UAE have also identified the presence of DcHEV-specific viral genomes in DCs where calves became infected during the first 6 months of life and cleared the virus with time^23^. In this study, the prevalence of HEV RNA was higher in domestic camels (4.1%) compared to imported camels (0.9%). The difference might be due to the younger age of domestic camels (the majority are 1–3 years old) where they acquire the infection early in life compared to the imported camels (all are 4–5 years old) where they have most probably acquired and cleared the DcHEV infection. Alternatively, this higher prevalence might indicate a possible risk factor for the infection in domestic camels responsible for this higher prevalence. In a recent study on the same subset of samples^25^, we showed that the seroprevalence of DcHEV in domestic camels is slightly higher than those of imported camels (25.4% *vs*. 22.4%, *p* value = 0.3). Observations from both studies warrant the need for further investigations to identify the possible risk factor that is causing this higher prevalence.

Both domestic and imported sequences clustered away from isolates reported from Pakistan. This indicates the presence of a genetic difference between sequences from this study and those from Pakistan. Further investigation is needed to clarify the significance of these differences. Phylogenetic analysis of the RdRp sequences in this study shows that they belong to HEV genotype 7. Sequences from domestic camels formed a separate cluster with reported sequences from Kenya, Somalia, and UAE (except for three samples) while imported sequences clustered with one isolate from UAE indicating a possible common source of infection. Previous studies have demonstrated the HEV seropositive DCs in Africa and the Middle East^21,25,34–37^. A recent study^37^ showed a higher seroprevalence in dromedaries in Israel where they found a seroprevalence of 68.6%. Further investigation is needed to identify the clinical role of these amino acid changes in the course of the disease. The duration seropositivity and the protective immune mechanisms also need to be defined.

Our data should be viewed considering several limitations of our study. Whilst our DCs study sample was large, the number of positive DcHEV cases was small. The low number of domestic camels compared to imported camels could not allow for statistical significance in the differences between the two groups of camels. Pooling leads to sample dilution which might have affected prevalence. The prevalence of HEV RNA might be underestimated because lifespan of viraemia in serum is short^23^. Study of more full genomes would have shown better the virus evolution, and future studies are suggested.

Our results provide evidence for the presence of DcHEV in both domestic and imported camels from Africa with evolving genomic changes. Further surveillance studies are required to identify the role of the identified DcHEV amino acid changes in the course of the disease. Our data highlight the potential role of DcHEV in the burden of HEV in the Saudi population specially in populations who have frequent contact with dromedaries like camel handlers and slaughterhouse workers and those who frequently consume camel meat and milk. Any change in transmissibility and virulence of DcHEV in DCs would affect the risks of spread to humans and its potential impact on national and regional public health security. Further clinical, serological, molecular and epidemiology investigations of DcHEV in human populations in the Middle East and Africa are needed to define and monitor the potential threat of DcHEV to Middle eastern and global public health security in light of 10 million pilgrims who visit Saudi Arabia every year from 182 countries.

## Supplementary Information

Supplementary Information.

## Data Availability

Data collected for the study, on camels, and molecular analyses can be made available upon request from the corresponding author.
