# Supplementary material for: Genetic diversity of hepatitis E virus (HEV) in imported and domestic camels in Saudi Arabia
Source: Sci Rep. 2022 Apr 29;12:7005. doi: 10.1038/s41598-022-11208-6 (PMC9054814; doi:10.1038/s41598-022-11208-6)
Supplement: Supplementary file 1 — Supplementary Information. [file 41598_2022_11208_MOESM1_ESM.docx]

Supplementary Table 1: Ct values for the positive samples tested by real time RT-PCR.

| **#** | **Sample No.** | **RT-PCR Ct.** | **Remark** |
| --- | --- | --- | --- |
| 1 | DC331 | 19.21 | Sequence Completed |
| 2 | DC333 | 22.3 | Sequence Completed |
| 3 | DC364 | 18.65 | Sequence Completed |
| 4 | 16S | 23.7 | Sequence Completed |
| 5 | DC446 | 26.87 | Sequence Completed |
| 6 | DC450 | 23.1 | Sequence Completed |
| 7 | DC470 | 22 | Sequence Completed |
| 8 | DC503 | 24.9 | Sequence Completed |
| 9 | DC506 | 32.7 | Sample was not sequenced because of its high Ct value |
| 10 | DC475 | 30.2 | Sequence Completed |
| 11 | DC12 | 22.87 | Sequence Completed |
| 12 | DC257 | 21.36 | Sequence Completed |
| 13 | 25S | 19.68 | Sequence Completed |
| 14 | 15S | 28.72 | Sequence Completed |
| 15 | 4S | 34.5 | Sample was not sequenced because of its high Ct value |
| 16 | 16SB | 27.42 | Sequence Completed |
| 17 | BS22 | 25.12 | Sequence Completed |
| 18 | MK791 | 19.23 | Sequence Completed |
| 19 | S9 | 24.63 | Sequence Completed |
| 20 | C934S | 21.59 | Sequence Completed |
| 21 | 362 | 29.51 | Sequence Completed |

Supplementary Table 2: Frequency distribution of the non-synonymous mutations detected in the RdRp region of the HEV viral genome in domestic and imported camels, sequences were aligned comparatively with the KJ496143 genotype 7 reference sequence.

| # | Nt Change | AA Change | Codon Change | AA position | Frequency in Domestic camels | Freqyency in Imported Camels |
| --- | --- | --- | --- | --- | --- | --- |
| 1 | A-T | Y-F | UAU-UTU | 32 | 2/10 (20%) | 0/9 (0%) |
| 2 | T-A | S-T | UCC-ACC | 110 | 6/10 (60%) | 2/9 (22.2%) |
| 3 | C-T | A-V | GCC-GTC | 40 | 0/10 (0%) | 1/9 (11.1%) |
| 4 | C-T | A-V | GCC-GTC | 92 | 2/10 (20%) | 0/9 (0%) |
| 5 | G-A | A-T | GCU-ACU | 46 | 1/10 (10%) | 0/9 (0%) |
| 6 | C-G | A-G | GCU-GGU | 29 | 2/10 (20%) | 0/9 (0%) |

**Supplementary Table 3:**

**Non-synonymous nucleotide changes in the full genomes of the DcHEV sequences of this study compared to the reference sequence KJ496143-HEV-7.**

| **Sample ID** | **Protein** | **AA Change** | **AA Number** | **Nt Change** | **Codon Change** |
| --- | --- | --- | --- | --- | --- |
| 362 | nonstructural polyprotein | D-E | 148 | C-A | GAC-GAA |
| 362 | nonstructural polyprotein | E-D | 489 | A-C | GAA-GAC |
| 362 | nonstructural polyprotein | L-P | 509 | T-C | CUU-CCU |
| 16s, 362 | nonstructural polyprotein | I-T | 539 | T-C | AUC-ACC |
| 16s | nonstructural polyprotein | P-S | 614 | C-T | CCU-TCU |
| 16s | nonstructural polyprotein | S-N | 624 | G-A | AGU-AAU |
| 362 | nonstructural polyprotein | M-I | 726 | G-A | AUG-AUA |
| 16s | nonstructural polyprotein | P-S | 735 | C-T | CCC-TCC |
| 16s | nonstructural polyprotein | E-G | 757 | A-G | GAG-GGG |
| 362 | nonstructural polyprotein | A-V | 760 | C-T | GCA-GTA |
| 16s, 362 | nonstructural polyprotein | A-P | 1123 | G-C | GCC-CCC |
| 16s, 362 | nonstructural polyprotein | R-Q | 1429 | G-A | CGG-CAG |
| 16s, 362 | nonstructural polyprotein | V-I | 1694 | G-A | GUC-AUC |
| 16s | capsid protein | M-I | 1 | G-A | AUG-AUA |
| 16s | hypothetical protein | C-Y | 5 | G-A | UGC-UAC |
| 16s, 362 | capsid protein | L-F | 13 | C-T | CUU-TUU |
| 16s | hypothetical protein | P-L | 74 | C-T | CCA-CTA |
| 16s, 362 | capsid protein | A-T | 80 | G-A | GCC-ACC |
| 362 | hypothetical protein | T-I | 93 | C-T | ACC-ATC |
| 16s | hypothetical protein | N-S | 94 | A-G | AAU-AGU |
| 16s, 362 | capsid protein | S-P | 97 | T-C | UCC-CCC |
| 362 | capsid protein | S-P | 105 | T-C | UCC-CCC |
| 362 | capsid protein | L-F | 383 | C-T | CUC-TUC |
| 16s | capsid protein | V-I | 538 | G-A | GUC-AUC |

Supplementary Figure 1: Percent similarity between the sequenced samples in the RdRp region of the HEV viral genome.

Samples starting with DC represent domestic camels. Color codes represent percentile similarity with red color indicating the highest similarity, yellow color indicating medium similarity and the green color indicating the lowest similarity between sequences.
